# Comparison of anticipated and actual control group outcome in randomised trials in paediatric oncology provides evidence that non-randomised studies are biased in favour of the novel treatment

**DOI:** 10.1186/1745-6215-12-S1-A108

**Published:** 2011-12-13

**Authors:** Veronica Moroz, Jayne Wilson, Keith Wheatley

**Affiliations:** 1 Cancer Research UK Clinical Trials Unit, University of Birmingham, UK

## Introduction

Historically controlled studies (HCSs) compare data from two or more separately conducted studies – the new treatment arm, usually prospectively collected, is compared to a control arm of retrospective data. HCSs are frequently undertaken in paediatric oncology (PO), where there is a widespread belief that RCTs cannot be performed in rare diseases. This is despite the potential biases in HCSs being well known – e.g. other factors change over time.

## Aim

To compare the outcome of the control group of RCTs in PO with that anticipated in the sample size calculation. The assumption being that, had an HCS been carried out instead, the control arm data in the historical control study would have likely been that utilized in the RCT’s sample size calculation.

## Methods

A search for published PO RCTs was conducted using the Cochrane Central database from March 2011 to database inception. Search terms were “randomi?ed” plus the disease name in all fields. Only papers reporting sample size parameters and observed estimates were included. Data were extracted and compared on anticipated and observed outcomes in the control arms (and experimental arms).

## Preliminary results

To date 45 RCTs have been included from 16 tumour sites: 36 superiority trials, 9 equivalence/non-inferiority; 12 with dichotomous primary endpoints, 33 time-to-event; mean recruited number of patients was 231 (range: 50 to 2619). In 33 trials the control group did better than anticipated (in 13 cases >10% absolute difference), in 11 trials the control group did worse (by >10% in four); outcome was the same as anticipated in one trial. The median absolute difference between control groups’ observed outcome and anticipated outcome was 5% (range: -21% to +35%); mean difference was also 5%. In superiority trials, the median difference was 6%; it was 4% in equivalence/non-inferiority trials. In trials with a dichotomous endpoint, the median difference was 7%; it was 5% in trials with a time-to-event endpoint (8 out of 9 equivalence/non-inferiority trials had time-to-event endpoint). The median observed difference between the experimental and control groups was 0% (range: -16% to 21%); the median difference between the observed experimental arm outcome and anticipated control group outcome was 6% (range: -20% to 45%).

## Interpretation

Since the control group (i.e. standard treatment arm) in RCTs did better than anticipated, non-randomised HSCs that use similar assumptions for outcome with standard treatment will overestimate the benefit of new treatments, potentially leading to children with cancer being given inappropriate therapy.

